# Fertility knowledge and family planning perspectives among female physicians: a cross-sectional study

**DOI:** 10.1007/s10815-026-03947-6

**Published:** 2026-07-02

**Authors:** Amelie Gonder, Cordula Schippert, Frauke von Versen

**Affiliations:** 1 Department of Obstetrics and Gynecology, Hannover Medical School, Hannover, Germany; 2 Department of Reproductive Medicine and Gynaecological Endocrinology, University Hospital Basel, University of Basel, Basel, Switzerland

**Keywords:** Fertility, Female physicians, Children and career, Family planning, Awareness

## Abstract

**Purpose:**

Female physicians spend a substantial proportion of their fertile years in academic and clinical training. The present study assessed fertility-related knowledge, concerns, and barriers to family planning among female physicians in Germany and their potential impact on career development.

**Material and methods:**

In this cross-sectional study, cisgender female physicians from all disciplines and career stages practicing in Germany were recruited via online platforms, printed flyers, and women physicians’ associations. An anonymous online survey was conducted between August 2024 and September 2025. It collected data on demographics, clinical, and academic career trajectories, fertility knowledge (using a modified FIT-KS and classified according to Bloom’s cut-off values), as well as motherhood and career intentions. Associations were examined using Pearson and Spearman correlation coefficients and chi-square tests. *p*-values were interpreted descriptively, with a significant threshold set at *p* < 0.05.

**Results:**

Of the 250 participating physicians, 214 completed the survey. The median age was 36.5 years (IQR 32–44). Respondents included residents (43.8%), specialists (22.3%), and senior physicians (19.5%). Overall fertility knowledge was moderate with a mean correct response rate of 70.2%. A total of 9.2% participants aspired to the highest academic rank, while 10.7% aimed for the highest clinical leadership position. Most respondents (90.2%) considered leadership positions compatible with part-time work. More than half reported postponing family planning, primarily due to limited career opportunities, long and inflexible working hours, and inadequate childcare support. Overall, 59% were mothers, with a mean of 1.99 children, and the mean age at first childbirth was 31.8 years. A higher age at first childbirth was significantly associated with retrospective regret about delaying parenthood. Physicians with children were more likely to work part-time. Among childless respondents, the main reasons were lack of suitable time due to studies or work (60.2%) and prioritization of career (45.5%).

**Conclusions:**

Fertility knowledge was moderate, suggesting that additional education may be needed to support informed family planning decisions. Greater acceptance and implementation of flexible working models, together with improved childcare infrastructure, may contribute to reducing barriers to balancing family and career.

**Supplementary Information:**

The online version contains supplementary material available at https://doi.org/10.1007/s10815-026-03947-6.

## Introduction

Female physicians spend a substantial proportion of their reproductive years in medical school, residency, and early career development. Compared with women in other educational groups, they tend to have their first child later in life, which can lead to fewer children or fertility-related problems [1–4].

As female fertility declines with advancing age, this delay places many physicians at increased risk of involuntary childlessness or subfertility, resulting in a higher utilization of fertility treatments [4, 5].

In Germany, the medical profession has seen a substantial increase in the proportion of women. According to the German Medical Association, women constituted just over 50% of practicing physicians in 2024 [6]. Despite this numerical parity, pronounced gender disparities persist across medical specialties, qualification levels, and leadership positions. Women remain markedly underrepresented in senior academic and executive roles, particularly at university hospitals, where only a small fraction of department heads and professors are female [7–10]. International and national studies consistently identify motherhood, pregnancy-related career interruptions, and unequal distribution of caregiving responsibilities as major contributors to these disparities [11, 12].

Academic medicine poses unique challenges for family planning. Long training periods, rigid working structures, limited childcare availability, and high-performance expectations often coincide with the biologically optimal window for reproduction [13, 14]. As a result, many female physicians postpone childbearing, frequently reporting later regret and a perceived incompatibility between career advancement and family life [11, 13].

Despite their medical training, several studies suggest that physicians—particularly trainees—may have incomplete or inaccurate knowledge about age-related fertility decline and reproductive limits [15]. Fertility awareness is a key determinant of reproductive decision-making, and misconceptions may contribute to delayed family planning and unrealistic expectations regarding assisted reproductive technologies [16, 17]. However, data on fertility knowledge and reproductive attitudes among female physicians in Germany are scarce. Against this background, the present study aimed to assess fertility knowledge, family planning experiences, and career-related challenges among female physicians in Germany. Using an exploratory cross-sectional design, we sought to identify patterns and associations related to childbearing timing, fertility awareness, career stage, work models, and perceived compatibility of family and professional life. By doing so, this study aims to contribute empirical data to an ongoing discussion on gender equity, workforce sustainability, and structural reform in academic and clinical medicine.

## Materials and methods

### Study design and setting

This cross-sectional study was conducted using an anonymous online questionnaire developed in German and administered with SoSci Survey (version 3.6.09). The study was approved by the Ethics Committee of Hannover Medical School (reference no. 11073_BO_K_2023). All participants provided informed consent, and the study was conducted in accordance with the Declaration of Helsinki. The questionnaire was publicly accessible from August 12, 2024, to September 30, 2025 (415 days).

### Primary and secondary outcomes

The primary outcome was fertility knowledge, assessed using a slightly modified version of the Fertility and Infertility Treatment Knowledge Score (FIT-KS) and categorized according to Bloom’s cut-off values.

Secondary outcomes included physicians’ perspectives on family planning, including factors influencing decision-making and timing. Physician age was analyzed in relation to age at first childbirth, consideration of social freezing, and work–family compatibility. The association between age at first childbirth and postponement of family planning with retrospective regret regarding timing of parenthood was also examined. In addition, the number of children was evaluated in relation to working model and extent of part-time employment. Childcare-related challenges were assessed with respect to work–family compatibility. Surgical specialties were further compared with other disciplines regarding rates of childlessness, number of children, and perceived work–family compatibility.

### Participants

To be eligible for participation in the survey, physicians had to meet the following inclusion criteria: they had to be cisgender female, hold a valid medical license, and be currently working as a physician in Germany. Consequently, individuals of other genders, medical students, nonphysician healthcare personnel, and those not currently employed as physicians in Germany were excluded from the study. If any of the inclusion criteria were not met at the beginning of the questionnaire, the survey was terminated automatically. Recruitment was conducted through multiple channels, including digital platforms and printed flyers, and was further supported by the German Medical Women’s Association (“Deutscher Ärztinnenbund e.V.”) and The Women Surgeons’ Association (“Die Chirurginnen e.V.”), both of which distributed the survey invitation to their members via email and encouraged participation.

### Questionnaire

#### Pretest

To assess question validity and response consistency, a pretest with a test–retest design was conducted using a 48-h interval. Participants were female licensed physicians, primarily employed at Hannover Medical School (MHH). Thirty physicians completed the initial test, and ten participated in the retest. Participant feedback was incorporated, resulting in minor revisions to the questionnaire. The revised version was subsequently used in a pilot project at MHH, where data were collected from 83 physicians.

#### Design

The final questionnaire comprised 64 items and required approximately 10–15 min to complete. Questions were either newly developed for this study or adapted from previously validated instruments [15]. A variety of response formats were used, including single and multiple choice, open-text responses, dropdown selections, and interval-scaled Likert items. For potentially sensitive questions, a “prefer not to answer” option was provided, and open-text fields (“other”) were included where predefined response options were insufficient. Filter questions were used to ensure that subgroup-specific items were displayed only to eligible participants. The most relevant stratification was by participants who already have at least one child and participants who do not have children.

After consent and eligibility screening, demographic and descriptive characteristics were collected (9 items), followed by sections on academic teaching and research (6 items), personal fertility (4 items), fertility knowledge (14 items), desire for children, pregnancy, and children (20 items), and future planning (8 items). An optional open-text field for comments was provided at the end of the questionnaire.

#### Fertility knowledge assessment

Fertility knowledge was partly assessed using adapted items from the Fertility & Infertility Treatment Knowledge Score (FIT-KS), a validated instrument designed to measure fertility awareness and knowledge of infertility treatments [15]. Selected FIT-KS items were modified to reflect the German context and updated epidemiological data; not all original items were included due to differences in national data and healthcare systems.

### Statistical analysis

An a priori sample size calculation indicated that 85 participants were required to detect a Pearson correlation of *r* = 0.30 with a two-sided *α* = 0.05 and 80% power. To account for multiple testing across 11 variable pairs, a conservative Bonferroni correction was applied (*α*′ = 0.05/11), increasing the required sample size to approximately 145. Allowing for incomplete responses, a target sample size of 200 participants was set. Data analysis was exploratory and descriptive. Pearson correlations were used for metric, Spearman’s rank correlations for ordinal variables or mixed metric-ordinal, and chi-square tests for nominal variables, with Fisher’s exact test applied to 2 × 2 tables.

A *p*-value < 0.05 was considered statistically significant; however, given the observational cross-sectional design, *p*-values are interpreted descriptively and not as confirmatory evidence. Knowledge scores were calculated as percentages and categorized using Bloom’s cut-off values (< 60% low, 60–80% moderate, and > 80–100% high) [18]. All analyses were performed using IBM SPSS Statistics version 30.0.0.0 (171).

## Results

### Participants and characteristics

A total of 250 datasets from physicians meeting all inclusion criteria were analyzed, of which 214 questionnaires were completed in full. Demographic and profession-specific characteristics of the study population are summarized in Table 1.

**Table 1:** Demographic characteristics

Characteristics	%	*n*
**Age in years** (*n* = 250)		
≤ 30	18.0%	45
31–35	26.8%	67
36–40	20.4%	51
41–45	13.6%	34
46–50	8.4%	21
51–55	5.6%	14
56–60	3.6%	9
61–65	3.6%	9
**Ethnicity** (*n* = 249)		
African	0.4%	1
Asian	1.6%	4
European	96.8%	242
Latin American	0.0%	0
Middle Eastern	0.8%	2
Indigenous	0.0%	0
Other ethnicity	0.0%	0
**Partnership**^#^ (*n* = 248)		
Single	10.5%	26
Married	55.2%	137
Monogamous relationship	32.3%	80
Open relationship	0.4%	1
Polyamory relationship	0.0%	0
Divorced	2.0%	5
Widowed	0.4%	1
Other relationship	0.4%	1
**Age at the start of medical school** (*n* = 246)		
≤ 19	52.8%	130
20–25	41.0%	101
26–30	5.2%	13
≥ 30	0.8%	2
**Current employer/workplace** (*n* = 245)		
University hospital	51.0%	125
Maximum care hospital (700 ≥ 1000 beds)	6.1%	15
Central and standard care hospital (300–699 beds)	17.9%	44
Minimum and basic care hospital (150–299 beds)	7.3%	18
Outpatient facility or doctor’s office (employed)	6.1%	15
Outpatient facility or doctor’s office (self-employed)	6.5%	16
Public institution, e.g., health department	2.4%	6
Another workplace	2.4%	6
**Medical disciplines** (*n* = 243)		
Surgery (all subgroups):	**23.0%**	**56**
General surgery	1.6%	4
Orthopedics and trauma surgery	9.5%	23
Pediatric surgery	1.6%	4
Visceral surgery	4.5%	11
Other surgery*	5.7%	14
General medicine	2.9%	7
Anesthesiology	8.2%	20
Ophthalmology	3.3%	8
Occupational medicine	0.4%	1
Dermatology	2.9%	7
Gynecology and obstetrics **	10.7%	26
ENT medicine	0.4%	1
Hygiene and environmental medicine	0.4%	1
Human genetics	0.4%	1
Internal medicine (all subgroups) ***	13.2%	32
Pediatrics ****	12.8%	31
Child and adolescent psychiatry	0.4%	1
Neurology	3.7%	9
Public health	0.8%	2
Pathology	0.4%	1
Clinical pharmacology	0.8%	2
Phoniatrics and pediatric audiology	0.4%	1
Physical and rehabilitation medicine	0.8%	2
Psychiatry	2.5%	6
Psychosomatic medicine	2.5%	6
Radiology *****	2.1%	5
Forensic medicine	0.4%	1
Urology	0.8%	2
Transfusion medicine	2.1%	5
Other disciplines	3.7%	9
**Current position** (*n* = 242)		
Resident physician	43.8%	106
Specialist physician	22.3%	54
Senior physician	19.54%	47
Leading senior physician	5.8%	14
Head of department, university professor	1.2%	3
Hospital director or chief physician	1.2%	3
Employed resident physician (outpatient care)	0.8%	2
Employed specialist physician (outpatient care)	2.1%	5
Employed specialist at an institution	1.2%	3
Practice owner	6.2%	15
Physician in a research institution	1.7%	4
Other function	2.1%	5
**Academic qualifications** (*n* = 239)		
No doctorate/PhD	25.5%	61
Doctorate/PhD	64.8%	162
Private lecturer (habilitation)	2.1%	5
Adjunct professor	2.5%	6
University professor	0.4%	1
Other qualification	1.7%	4
**Full-/part-time** (*n* = 241)		
Full-time	62.2%	150
Part-time	37.8%	91
**Working time in percent of part-time employees** (*n* = 91)		
< 25%	1.1%	1
25–50%	7.7%	7
50–75%	42.9%	39
75 ≤ 100%	48.4%	44
**Do you currently have children, and if so, how many?** (*n* = 217)		
I don’t have any children	41.0%	89
1	17.5%	38
2	27.2%	59
3	11.5%	25
4	2.8%	6
**Experienced miscarriages** (*n* = 217)		
0	75.6%	164
1	13.4%	29
2	6.9%	15
> 2	3.7%	8
Don't want to answer	0.5%	1

^**#**^**Multiple choice question**

*Other surgery: vascular surgery, cardiac surgery, neurosurgery, oral and maxillofacial surgery, plastic-reconstructive-aesthetic-surgery, thoracic surgery

**Incl. gynecological endocrinology, reproductive medicine and gynecology, gynecologic oncology, special obstetrics and prenatal medicine

***Incl. angiology, endocrinology and diabetology, gastroenterology, hematology and oncology, cardiology, nephrology, pulmonology, rheumatology

****Incl. pediatric cardiology, neonatology, neuropediatric

*****Incl. pediatric radiology, neuroradiology

#### Fertility knowledge (n217)

**Fig. 1:**
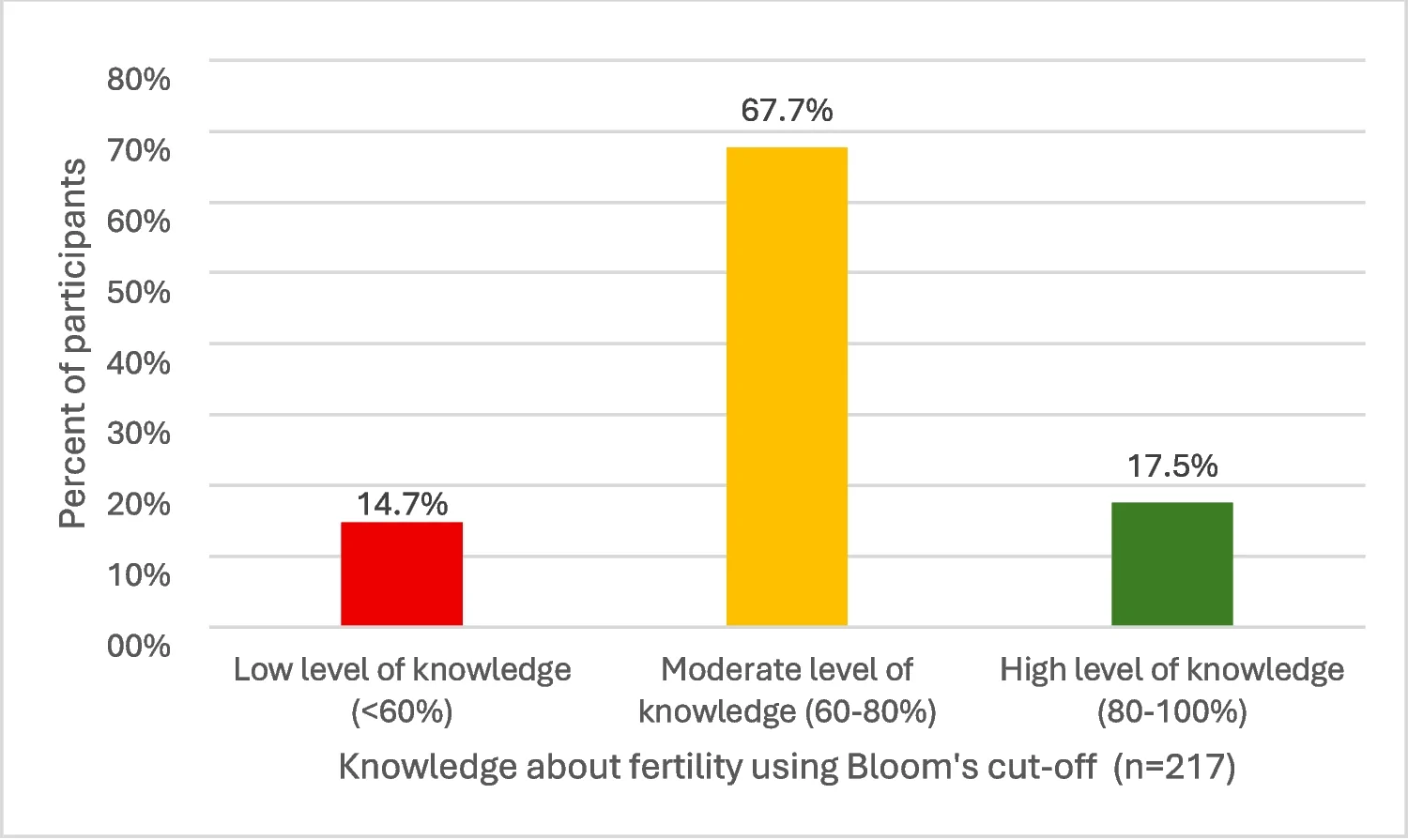
Distribution of Bloom’s cut-off levels indicating knowledge about fertility (*n* = 217)

Prior to objective assessment participants self-rated their fertility knowledge as good (44.0%) or very good (20.9%), while 26.1% considered it sufficient and 9% inadequate. Most participants agreed that fertility education should be expanded (61.6%), whereas 36.1% expressed a neutral view. The results for the primary outcome, fertility knowledge, showed correct response rates ranging from 40.9 to 90.9%. The mean score of 70.8% corresponded to a moderate level of fertility knowledge according to Bloom’s cut-off values [15, 18]. The results are shown in Fig. 1.

While most participants correctly identified the most fertile age range (90.9%) and the optimal timing of intercourse (98.7%), substantial knowledge gaps were observed regarding age-related fertility decline, monthly conception probabilities, miscarriage risk, sperm survival time, and modifiable lifestyle factors. Fertility was universally recognized as involving both partners, although nearly one-fifth underestimated the role of male age. Misconceptions were particularly common concerning the effects of moderate alcohol consumption, prior oral contraceptive use, and fertility-affecting lubricants. Detailed results are provided in Table 2.

**Table 2:** Assessment of fertility knowledge among participants

1. At what age are women most fertile? (n = 231)	%	*n*
12–19	9.1%	21
20–29*	**90.9%**	**210**
30–39	0.0%	0
40–49	0.0%	0
2. Over which age range does a woman’s ability to get pregnant decline most precipitously? (*n* = 231)		
20–25	0.4%	1
30–34	13.9%	32
35–39*	**58.0%**	**134**
40–45	27.7%	64
3. Over the course of 1 month, what is the percent chance that a 30 years old woman who is trying to get pregnant will get pregnant? (*n* = 231)		
10%	22.5%	52
20%*	**43.3%**	**100**
30%	29.4%	68
40%	4.8%	11
4. Over the course of 1 month, what is the percent chance that a 40 years old woman who is trying to get pregnant will get pregnant? (*n* = 231)		
≤ 5%*	**65.8%**	**152**
6–10%	29.0%	67
11–15%	3.5%	8
16–20%	1.7%	4
5. On average, for a woman in her peak reproductive years, what is the percent chance that a pregnancy (recognized or unrecognized) will end in a miscarriage? (*n* = 231)		
< 5%	6.9%	16
6–15%	40.3%	93
16–25%*	**32.0%**	**74**
26–35%	20.8%	48
6. A woman and a man can both contribute to a couple's infertility: (*n* = 231)		
True*	**100%**	**231**
False	0.0%	0
7. A man’s age is a factor that affects a couple’s fertility: (*n* = 229)		
True*	**83.0%**	**190**
False	17.0%	39
8. What is the average survival time of normal sperm in the female reproductive tract? (*n* = 227)		
12–24 h	11.9%	27
24–48 h	47.6%	108
3–5 d*	**38.3%**	**87**
6–9 d	2.2%	5
9. When is the optimal time to have sexual intercourse in order to get pregnant? (*n* = 225)		
Right before the period starts	1.3%	3
First few days of the period	0.0%	0
About halfway through the cycle*	**98.7%**	**222**
It doesn’t matter	0.0%	0
10. Where does fertilization most commonly occur? (*n* = 224)		
In the uterus	20.1%	45
Inside the ovaries	4.0%	9
On the surface of the ovaries	3.6%	8
In the fallopian tubes*	**72.3%**	**162**
11. How many eggs are typically released per cycle? (*n* = 224)		
1*	**95.1%**	**213**
2	2.2%	5
3	1.3%	3
4	1.3%	3
12. The following are likely to decrease a woman’s chance of fertility: (*n* = 222)	Correct answer	
Smoking (true)	**100.0%**	222
Occasional caffeine intake (false)	**86.9%**	193
Moderate alcohol consumption (true)	**68.5%**	152
Safely conducted pregnancy termination (false)	**67.1%**	149
Obesity (true)	**97.3%**	216
Gonorrhea or chlamydia infection (true)	**100.0%**	222
Prior use of oral contraceptive pills (false)	**44.1%**	98
Being underweight due to frequent exercise or limited caloric intake (true)	**99.1%**	220
Using certain types of sexual lubricants (true)	**59.0%**	131
13. For a woman under 35 years old, undergoing IVF with her own eggs, what is the pregnancy rate per cycle? (*n* = 218)		
< 5%	4.1%	9
6–25%*	**51.8%**	**113**
25–45%	35.3%	77
45–60%	7.3%	16
> 60%	1.4%	3
14. For a woman over 44 years old, undergoing IVF with her own eggs, what is the pregnancy rate per cycle? (*n* = 217)		
< 5%*	**59.9%**	**130**
6–25%	36.4%	79
25–45%	3.2%	7
> 60%	0.5%	1

*Indicates the correct answers

## Desire to have children (n = 217)

In line with the age distribution of the sample, 40.6% of respondents indicated that they had already completed their family planning. However, 29.5% reported a current desire to have children and 12.9% leaned toward wanting children, whereas 11% leaned toward or clearly stated not wanting children; 4.6% were undecided. The desire to have children most frequently emerged during residency (51.6%), followed by medical school (14.7%). Few participants reported first experiencing this desire before medical school (4.1%) or during later career stages. Nearly half of respondents (48.7%) reported having previously been concerned about their own fertility, most commonly due to age (53.9%), stress (52.2%), and social pressure (20.9%). More than half of all respondents (54.8%), whether they already have children or not, stated that they had postponed family planning, mainly due to reduced career advancement opportunities (68.3%), long/inflexible working hours (65.8%), and inadequate childcare options (52.5%).

At the time of the survey, 41.0% (*n* = 89) of respondents were childless. Among childless respondents the most common reasons were lack of suitable time due to studies or work (60.2%) and prioritization of career (45.5%). Additional reasons included absence of a suitable partner (18.2%), financial uncertainties (15.9%), perceived age-related concerns, involuntary childlessness (18.2%), and lack of desire for children 10.2%; 3.4% reported being currently pregnant.

### Motherhood, parental leave, and compatibility of work and life

#### Participants with children (n = 128)

**Fig. 2:**
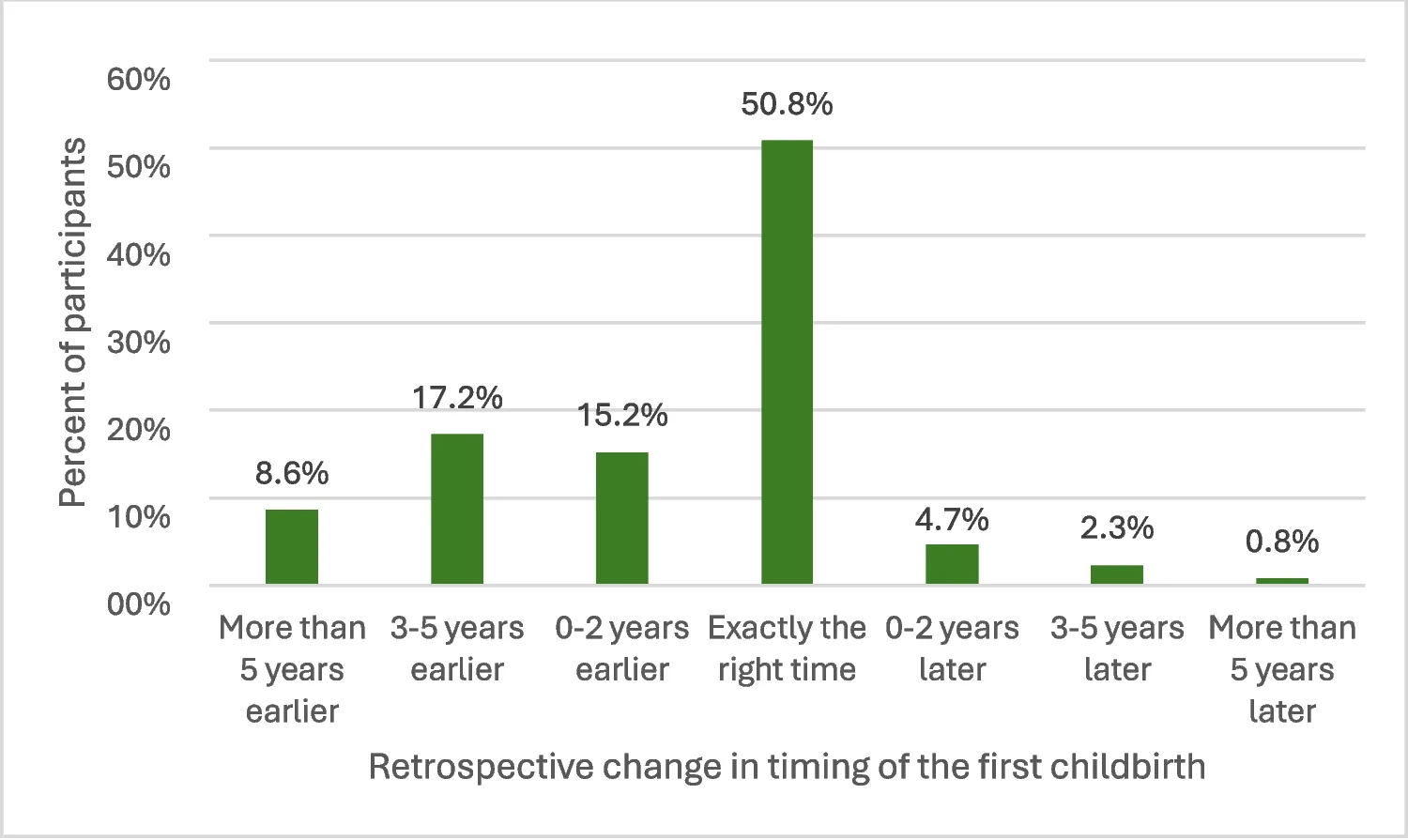
Retrospective participant perspectives on first childbirth timing (*n* = 128)

**Fig. 3:**
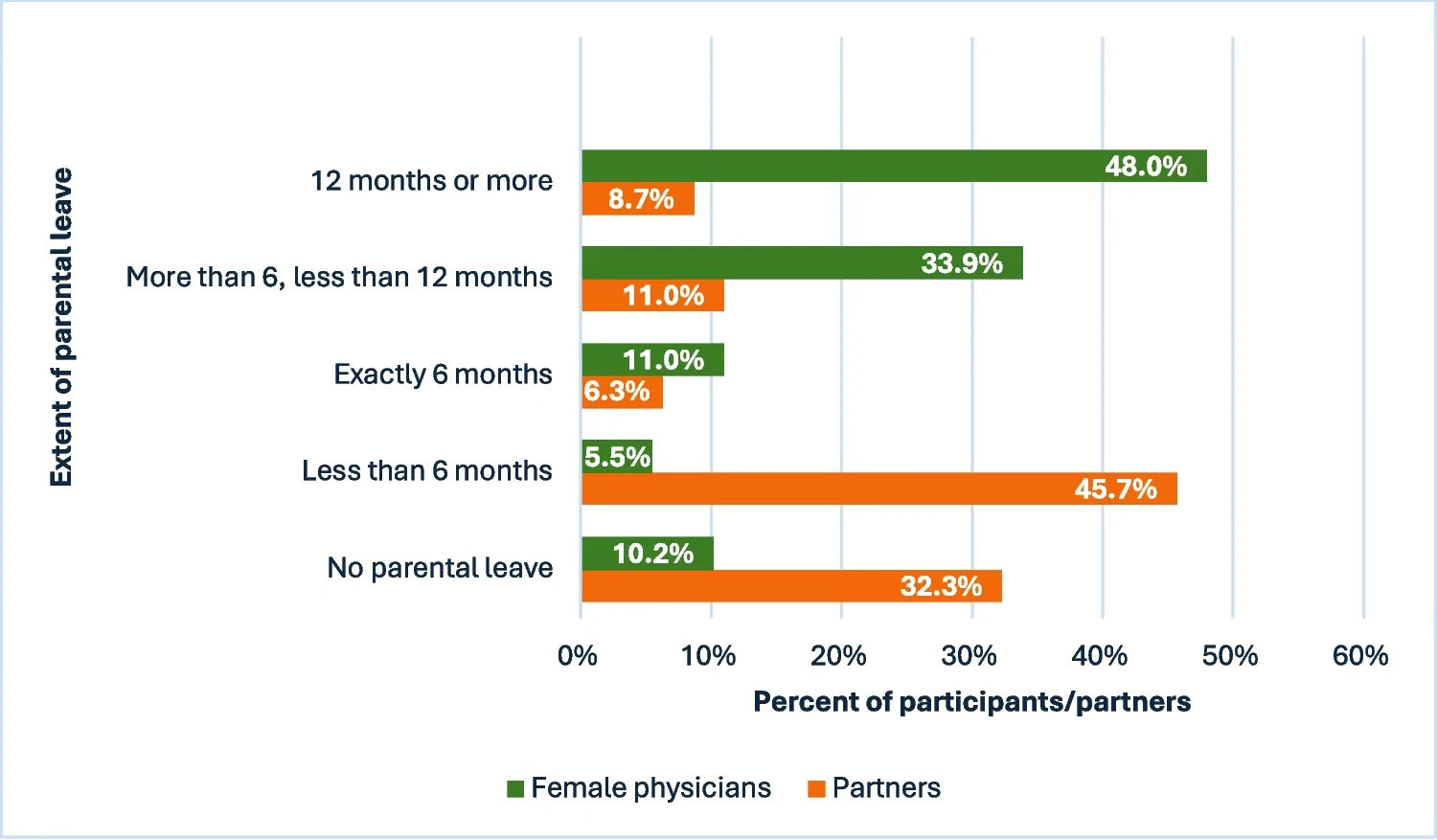
Comparison of the extent of parental leave taken by female physicians and their partners (*n* = 127)

At the time of the study, 59% of respondents had already at least one child, with an average of 1.99 children per mother. The mean age of participants in this subgroup was 31.8 years, ranging from 20 to 42 years. No significant correlation was found between the physicians’ age and the age at which they had their first child (*n* = 128, *r* = 0.159, *p* = 0.07), suggesting no significant generational shift in age at first childbirth. Most chose to have children during residency (57.0%) or as specialists (44.5%), with only 14.8% having children during medical studies and 1.6% before starting medical school. Almost half of the mothers surveyed would have chosen a different time to give birth to their first child in retrospect, Fig. 2. Postponing family planning was also significantly correlated with preferring a different timing (*n* = 128, chi^2^ = 19.512, *p* < 0.001). Likewise, there was a significant negative correlation between age at first childbirth and the desire for an earlier timing (*n* = 128, *ρ* =  − 0.596, *p* = 0.01). Factors influencing family planning included a supportive partner (83.5%), financial stability (67.7%), and a partner willing to take parental leave (41.7%). Less common factors were positive role models at work (25.2%) and a reliable childcare system (21.3%). Most of the mothers surveyed were dissatisfied with the compatibility of career and family life, with 74.0% reporting dissatisfaction, 11.0% neutral responses, and only 14.9% expressing satisfaction. Parental leave patterns varied between mothers and their partners. Among the mothers, 10.2% did not take parental leave, while nearly half (48.0%) took more than 12 months; 33.9% took 6–12 months and only a small proportion took 6 months (11.0%) or less than 6 months (5.5%), Fig. 3. In contrast, 32.3% of partners did not take parental leave, and most (45.7%) took less than 6 months. Only few partners took exactly 6 months (6.3%), more than 6 months (11.0%), or more than 12 months (8.7%), Fig. 3.

Compatibility of family and work was perceived negatively, with 69.3% of mothers reporting difficulties finding suitable childcare. State institutions were most commonly used (39.4%), followed by private childminders (32.3%); relatives, private, or church institutions were used less frequently, and au pair care was rare (4.7%).

Most respondents reported that their employer did not offer childcare or capacity was insufficient (37.2% and 37.7%, respectively), while only 5.1% reported sufficient employer-provided childcare. Overall, 37.8% of respondents worked part-time, which was significantly positive associated with the number of children (*n* = 217, *ρ* = 0.471, *p* < 0.001). Among part-time workers, most reduced their workload only moderately (> 75%, 48.4%; 50–75%, 42.9%), and the extent of part-time work was not significantly associated with the number of children (*n* = 88, *ρ* = 0.190, *p* = 0.08). Detailed results are presented in Additional file 1.

#### Female physicians in surgery (n = 51)

There was no significant difference in number of children between surgical and nonsurgical specialties (*n* = 217, chi^2^ = 2.209, *p* = 0.69). Physicians in surgical specialties were also not significantly more likely to be childless (*n* = 217, chi^2^ = 0.124, *p* = 0.72). There was also no significant correlation between surgical specialty and satisfaction with work–family balance in our sample (*n* = 127, chi^2^ = 0.390, *p* = 0.82)^*^.^1^

#### Fertility treatment and social freezing (n = 217)

Regarding fertility treatment, 15.7% of participants stated having undergone or currently undergoing fertility treatment, while the majority (83.9%) had never needed a fertility treatment. The cryopreservation of oocytes as fertility-preserving option (also called social freezing) appeared to be less common, with only 2.3% of respondents having created a fertility reserve, and 0.9% having used it. However, 12.8% considered social freezing as an option for fulfilling their desire to have children later, and 24.6% tended to see it as a viable option. The remaining respondents either had a neutral stance (10.4%) or did not consider it an option (52.1%).

Over the past decade, the perception of social freezing as a fertility preservation method has evolved. Younger physicians were more likely to consider social freezing. A significant negative association existed between respondent age and the consideration of social freezing (*n* = 211, *ρ* =  − 0.155, *p* = 0.03).

### Interest in academic teaching and research

Regarding further academic qualification (multiple responses allowed; *n* = 239), most participants did not intend to pursue further additional qualifications. Among those interested, 24.3% aimed for a doctorate/PhD, 17.6% for a private lectureship, 9.2% for a university professorship, 7.1% for an adjunct professorship, and 3.8% for another qualification. One-third of respondents (*n* = 238) were currently involved in academic teaching (33.6%), while 10.5% had participated previously and 24.4% planned future involvement, 31.5% expressed no interest. Similar patterns were observed for academic research (*n* = 236), with 29.2% of respondents currently involved, 13.1% previously involved, and 21.6% planning future engagement, while 36.0% expressed no interest.

The main reasons given in a multiple-choice question for currently not participating (*n* = 168) were, in both the research and teaching sectors, the prioritization of clinical work (research 62.5%; teaching 53.0%) and the considerable time commitment involved (research 44.6%; teaching 39.6%).

#### Career goals and future planning (n = 214)

Participant’s preferred long-term career settings were diverse, with university hospitals most popular (32.7%), followed by outpatient facilities or private practices (27.6%). Fewer chose minimum/basic care hospitals 4.7%, central/standard care hospitals (13.1%), or maximum care hospitals (7.0%), while 8.9% had no concrete plans.

Most respondents were confident that they would achieve their career goals, with 41.5% indicating it was likely and 25.1% very certain, while 10.8% considered it unlikely and 2.1% very unlikely.

Regarding career aspirations (multiple responses) the main reasons for not pursuing higher positions were current satisfaction (43.5%), and unwillingness to invest more time (21.7%), with fewer citing reluctance to take on responsibility (13.0%) or lack of interest. Age was not significantly correlated with work-family satisfaction (*n* = 127, *ρ* = 0.097, *p* = 0.28), but difficulties finding childcare were significantly associated with lower satisfaction (*n* = 127, chi^2^ = 11.924, *p* = 0.03).

#### Management position and discrimination (n = 214)

**Fig. 4:**
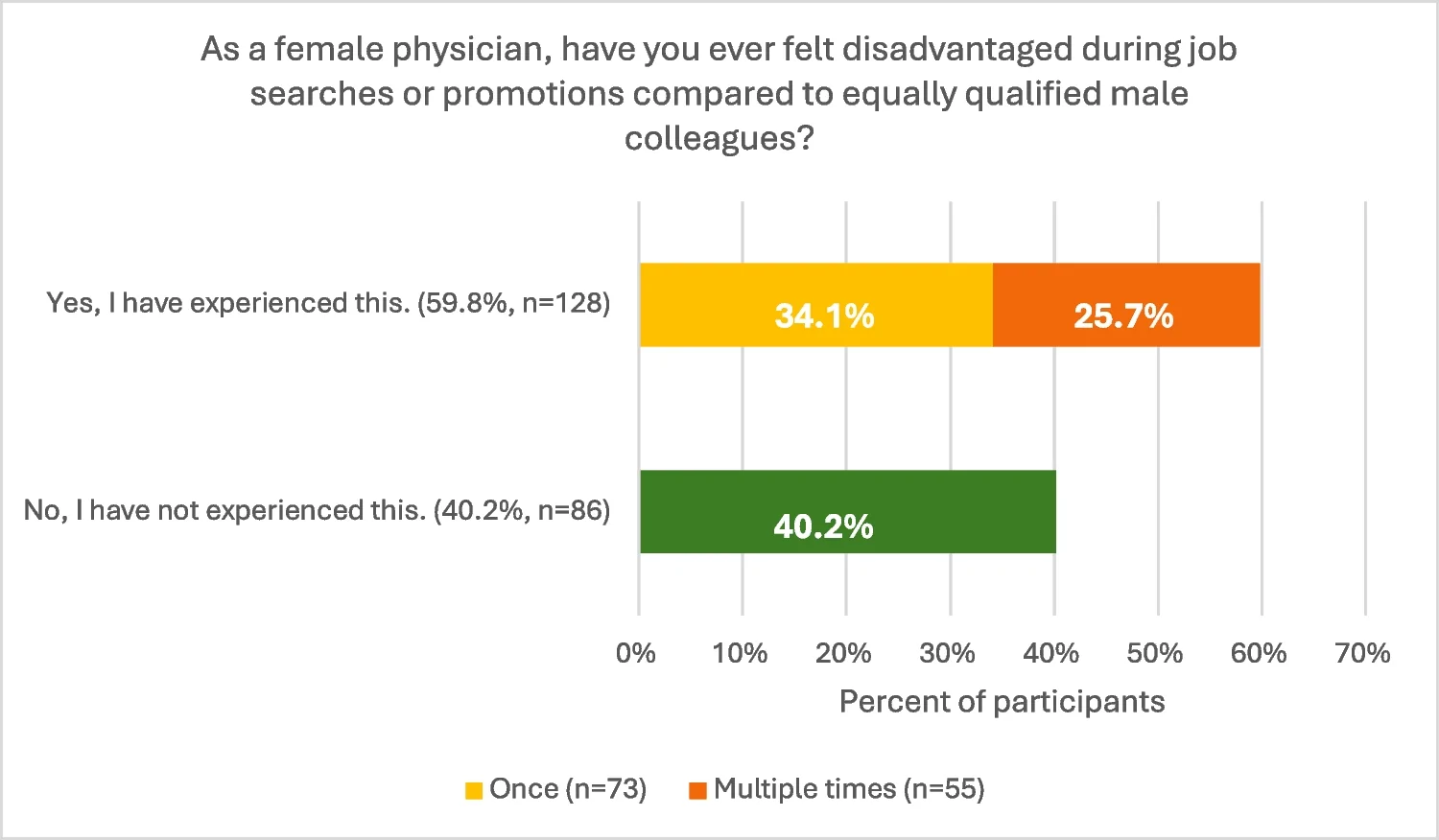
Discrimination compared to men with equal qualifications (*n* = 214)

Most participants (46.3%) believed that management positions are feasible with a 50% working week, while 34.6% considered a capacity of at least 75% to be necessary. A small number felt that this was only possible with a capacity of over 75% (7.0%), and very few stated that this was impossible on a part-time basis (5.1%), while 2.3% considered a 25% capacity to be sufficient and 4.7% were unsure. Among those who applied for a part-time management (*n* = 94), 23.4% had been rejected and 11.7% multiple times due to their part-time preference; 64.9% reported no such experience. Participants were also asked whether they had ever felt disadvantaged as female physicians in hiring or promotions (*n* = 214) with the results provided in Fig. 4.

## Discussion

This study provides a comprehensive overview of fertility knowledge, family planning, and career-related experiences among cisgender physicians in Germany. Several key findings emerge that align with, and extend, existing national and international literature.

Overall, fertility knowledge among participants was at a moderate level, as assessed by adapted FIT-KS items. While most respondents correctly identified general concepts such as most fertile age range and optimal timing of intercourse, substantial knowledge gaps were observed regarding age-related fertility decline, conception probabilities, miscarriage risk, and modifiable lifestyle factors. These findings are consistent with previous studies among medical trainees and physicians, which report persistent misconceptions despite medical education [19]. Given physicians’ dual roles as both potential patients and healthcare providers, these gaps underscore the need for structured fertility education within undergraduate and postgraduate medical curricula. To raise awareness of fertility issues among physicians, a multifaceted approach that goes beyond simply imparting knowledge could be helpful. In addition to incorporating fertility education into university training, offering targeted low-threshold information resources for everyday clinical practice, such as short e-learning modules could be offered [20, 21]. Also, integration into workplace health programs, on a voluntary basis and in accordance with data protection regulations, can be an option. There are platforms that employers can use to access information and services relating to fertility, though they are rarely offered in Germany [22, 23].

In line with earlier research, physicians in this study had fewer children and a higher age at first childbirth compared to the general population in Germany [24, 25]. The average number of children per participant (1.18) closely mirrors historical data for physicians and academics, indicating that this pattern has remained stable for decades despite broader societal changes [26]. Importantly, older age at first childbirth was associated with retrospective regret and a preference for earlier family formation, a finding also reported in prior qualitative and quantitative studies [13].

Our finding that lack of time due to studies or work, as well as the prioritization of clinical activities, is a major reason for postponing family planning, is consistent with previous studies showing that female doctors often delay starting a family due to the demands of medical training and career advancement [3]. Another important finding was that difficulties in finding childcare were significantly associated with lower satisfaction regarding work–life balance. Other studies have also shown that this is a structural problem affecting work-life balance [27]. This suggests that access to reliable childcare is not just a logistical issue, but a crucial factor influencing the perceived compatibility of a medical career and family life. The observation in this study that younger physicians increasingly consider fertility preservation options, such as social freezing, reflects growing awareness of reproductive aging. However, actual utilization remains low, which may be due to factors such as financial barriers, ethical concerns, and uncertainty about long-term outcome factors also reported in international literature [28, 29]. Our findings further highlight the enduring impact of motherhood on career trajectories in medicine. Female physicians disproportionally assume longer parental leave and are more likely to transition to part-time work, reinforcing documented gender asymmetries in unpaid care work [10, 12, 30]. This pattern persists despite high professional qualifications and contributes to reduced research output, slower promotion, and underrepresentation in leadership positions [7, 30, 31].

Consistent with previous German studies, many respondents reported having experienced disadvantage in hiring or promotion processes compared with equally qualified male colleagues. Although part-time leadership roles were widely considered feasible, such positions remain rare in practice. Innovative models such as top-sharing, which have shown high acceptance among senior physicians, may represent a viable strategy to improve gender equity while maintaining leadership continuity [32].

### Limitation and further research

This study has several limitations. First, the sample may not be fully representative of the broader population of physicians, as it is affected by selection bias. Recruitment challenges, particularly among female physicians across diverse specialties, and those working in peripheral healthcare settings, resulted in underrepresentation of these groups—especially general medicine practitioners [6]. In contrast, physicians working in university hospitals, often engaged in academic teaching and research, are disproportionately represented. Selection bias may also have been introduced through recruitment via an interest focused on fertility and gender equity, potentially attracting participants with a preexisting interest in this topic. In addition, the relatively young mean age of respondents further limits generalizability of the findings. The subgroup of women in surgery is particularly small, limiting the interpretability of the results for this population.

Future research could usefully compare physicians in inpatient versus outpatient settings to better understand differences in work-life balance. Including cisgender male physicians would also provide important context to gender-specific context. Moreover, same-sex relationships and broader aspects of gender diversity were not assessed, which may limit the applicability of the findings to sexual and gender minority physicians. Further perspectives should be considered, including potential differences in fertility knowledge. Due to multiple sources of selection bias, this study cannot be considered representative of a broad, objective cross-section of physicians in Germany. Its cross-sectional and exploratory design further precludes any causal inference. In addition, the partial adaptation of the FIT-KS questionnaire limits direct comparison with international studies.

## Conclusion

This study provides insight into fertility knowledge, family planning, and career development among cisgender female physicians in Germany. Participants showed moderate fertility knowledge with gaps regarding age-related fertility decline, suggesting that medical training does not necessarily translate into comprehensive reproductive knowledge. A structured integration and higher prioritization of fertility education into basic medical training curricula may improve both professional competence and personal reproductive awareness. Many respondents reported postponing family planning because of professional demands, while motherhood and part-time employment were associated with reduced career progression, highlighting persistent structural barriers. Addressing these challenges through flexible work arrangements, equitable parental leave, and improved childcare may promote gender equity, support retention in academic and leadership roles and contribute to reducing barriers to balancing family and career.

## Supplementary Information

Below is the link to the electronic supplementary material.

Supplementary Material 1 (DOCX 20.2 KB)

## Data Availability

The data supporting the findings of this study are available from the corresponding author upon reasonable request.
