# Supplementary material for: Fertility knowledge and family planning perspectives among female physicians: a cross-sectional study
Source: J Assist Reprod Genet. 2026 Jul 2;43(8):2409–21. doi: 10.1007/s10815-026-03947-6 (PMC13597974; doi:10.1007/s10815-026-03947-6)
Supplement: Supplementary file 1 [file 10815_2026_3947_MOESM1_ESM.docx]

# Additional file 1

Suppl. Table 1 Personal fertility, desire for children, pregnancy, fertility reserve

|  | % | n |
| --- | --- | --- |
| **Have you ever been concerned that your own fertility might be impaired? (n=236)** |  |  |
| No | 45.3% | 107 |
| Yes | 48.7% | 115 |
| I haven't given it any thought so far. | 5.9% | 14 |
| **Why were you concerned about your fertility?^#^ (n=115)** |  |  |
| Because of my age | 53.9% | 62 |
| Smoking | 0.9% | 1 |
| Obesity | 9.6% | 11 |
| Fitness | 6.1% | 7 |
| Stress | 52.2% | 60 |
| Diseases that could have an impact | 17.4% | 20 |
| Fertility problems in close relatives | 11.3% | 13 |
| Social pressure | 20.9% | 24 |
| Partner's fertility problems | 10.4% | 12 |
| Other | 16.5% | 19 |
| **Would you like to have children in the future? (n=217)** |  |  |
| My family planning is already complete. | 40.6% | 88 |
| No, I don't feel the desire to have children. | 6.5% | 14 |
| Rather not | 6.0% | 13 |
| Neutral | 4.6% | 10 |
| Rather yes | 12.9% | 28 |
| Yes | 29.5% | 64 |
| **When did you first feel a serious desire to have children? (n=217)** |  |  |
| Never | 8.3% | 18 |
| Before studying medicine | 4.1% | 9 |
| During medical studies | 14.7% | 32 |
| During the practical year | 4.6% | 10 |
| During my time as a resident | 51.6% | 112 |
| During my time as a specialist | 10.1% | 22 |
| During my time as a senior physician | 3.2% | 7 |
| I don't remember | 3.2% | 7 |
| **Have you postponed your family planning? (n=217)** |  |  |
| Yes | 54.8% | 119 |
| No | 45.2% | 98 |
| **Do you currently have children, and if so, how many? (n=217)** |  |  |
| I don't have any children. | 41.0% | 89 |
| 1 | 17.5% | 38 |
| 2 | 27.2% | 59 |
| 3 | 11.5% | 25 |
| 4 | 2.8% | 6 |
| **When did you have your child/children?^#^ (n=128)** |  |  |
| Before studying medicine | 1.6% | 2 |
| During medical studies | 14.8% | 19 |
| During my time as a resident | 57.0% | 73 |
| During my time as a specialist | 44.5% | 57 |
| Other time | 8.6% | 11 |
| **If you do not have children, what are the reasons for this? ^#^ (n=88)** |  |  |
| I never felt the desire to have children. | 12.5% | 11 |
| Despite wanting to have children, I have not yet become pregnant. | 18.2% | 16 |
| I feel too young. | 20.5% | 18 |
| I feel too old. | 9.1% | 8 |
| There is currently no suitable partner. | 18.2% | 16 |
| I have prioritized my career higher. | 45.5% | 40 |
| Financial uncertainties | 15.9% | 14 |
| Due to my studies and work, there hasn't been a suitable time yet. | 60.2% | 53 |
| I am currently pregnant. | 3.4% | 3 |
| Other | 9.1% | 8 |
| **Have you ever had fertility treatment or are you going through one? (n=217)** |  |  |
| No, I have never had fertility treatment. | 83.9% | 182 |
| Yes, I have already had fertility treatment. | 12.9% | 28 |
| Yes, I am currently undergoing fertility treatment. | 2.8% | 6 |
| I don't want to answer that. | 0.5% | 1 |
| **Have you or have you ever had a fertility reserve (freezing of eggs, "social freezing")? (n=217)** |  |  |
| Yes, I have built up a fertility reserve and have already used it. | 0.9% | 2 |
| Yes, I have built up a fertility reserve, but I haven't used it yet. | 1.4% | 3 |
| Yes, I have built up a fertility reserve, but I never required it. | 0.5% | 1 |
| No, I haven’t built up a fertility reserve. | 97.2% | 211 |
| **Can you imagine or could you have imagined creating a fertility reserve (freezing eggs, "social freezing") to fulfill possible desire to have children later? (n=211)** |  |  |
| No, that was/is not an option. | 28.9% | 61 |
| Not really | 23.2% | 49 |
| Neutral | 10.4% | 22 |
| Rather yes | 24.6% | 52 |
| Yes, that was/is an option. | 12.8% | 27 |

**^#^**Multiple choice question
